# Italian University Students’ Resilience during the COVID-19 Lockdown—A Structural Equation Model about the Relationship between Resilience, Emotion Regulation and Well-Being

**DOI:** 10.3390/ejihpe13020020

**Published:** 2023-01-21

**Authors:** Roberta Renati, Natale Salvatore Bonfiglio, Dolores Rollo

**Affiliations:** 1 Department of Pedagogy, Psychology, Philosophy, University of Cagliari, 09123 Cagliari, Italy; 2 Noah SRL, 27100 Pavia, Italy; 3 Department of Medicine and Surgery, University of Parma, 43121 Parma, Italy

**Keywords:** resilience, emotion regulation, pandemic, COVID-19, university students, emerging adults, distress

## Abstract

Over the past two years, the consequences of the severe restrictions imposed by the rapid spread of COVID-19 among the global population have been a central focus of scientific research. The pandemic has been a singular and unexpected event that found people unprepared and vulnerable in responding to its emergence, resulting in substantial psychological distress. Scientific evidence has highlighted that adolescents and emerging adults have been among those populations at greatest risk of adverse psychological outcomes, even in the long term. In particular, more than one-third of young adults reported high levels of loneliness, and nearly half of 18- to 24-year-olds felt lonely during the pandemic, experiencing both psychological and emotional distress. The lockdown, the consequent suspension of face-to-face academic activities and the severe restriction of social life have disrupted the daily routines of students already involved in coping with developmental tasks related to identity formation and the relational experience. Under such conditions, emotions and emotional regulation skills are crucial in adapting behavior to reach academic goals and face mounting levels of distress. Therefore, several studies have investigated resilience mechanisms and coping strategies of emerging adults during the pandemic. The present study focuses on university students and explores the impact of resilience and emotional regulation on adverse psychological outcomes related to persistent distress conditions associated with the COVID-19 pandemic. Students were administered a self-report assessment battery through an online platform at the beginning (T0) and the end of the lockdown (T1). A structural equation model (SEM) was used to explore the relationship between resilience, emotional regulation difficulties and psychological distress (depression, anxiety and stress). The findings indicate that psychological resilience and emotion regulation are protective factors that buffer the extent of possible distress resulting from an adverse condition such as the COVID-19 pandemic.

## 1. Introduction

At the beginning of March 2020, the 2019 coronavirus pandemic (COVID-19) was declared a significant global public health emergency. The rapid spread of SARS-CoV-2 has dramatically changed the lives of millions of people around the world. In Europe, Italy was the first country to be severely affected by the diffusion of the virus. To limit the spread of the infection, the government took protective and preventive measures, such as general lockdowns and curfews, which persisted for a prolonged period with adverse consequences on all productive sectors of society and, most impotantly, on people’s psychological health. In particular, the forced social distancing and the school and university closures have caused massive restrictions on human activities and physical interactions, leading to consequences on the population’s well-being, especially for students at all levels of the educational system. According to the United Nations Educational, Scientific and Cultural Organization (UNESCO), we are now facing a profound crisis in educational systems, including the universities. A large body of literature has pointed out that the restrictions imposed by the COVID-19 pandemic, such as lockdowns and the resulting suspension of face-to-face academic activities, have severely impacted students’ daily routines, affecting their well-being. The novel and complex scenario of the pandemic, coupled with feelings of uncertainty and fear, seems to have had a negative impact on the well-being of university students who, during the period of restrictions, have faced a landscape characterized by substantial uncertainty for their academic projects, but also concerning job perspectives and their personal and family life plans.

Psychological distress and emotional vulnerability have been considered negative consequences determined by the COVID-19 pandemic and related to distress variables such as anxiety, boredom, low life satisfaction and negative expectations toward the future [1,2]. Several studies have shown that psychological resilience has been one of the helpful protective factors in reducing the negative consequences of the pandemic. In a review, Serafini et al. [2] collected several studies that point out that among the first psychological responses to an event of such magnitude are increased anxiety, distress, mood alteration and irritability. Later, frustration and boredom also come into play, which, together with social isolation due to lockdowns, can lead to a crippling sense of loneliness. Moreover, Chu et al. [1], in a systematic review about the consequences of the pandemic, identified several social areas affected by the pandemic: psychological distress, high inequalities in communication, economic difficulties, reduced accessibility to health care systems, increased violence and gender inequality and instability in educational provision. Several studies have identified that the most at-risk populations, after health workers, are children, adolescents and young adults [1,2,3,4,5,6,7]. More than others, these three population groups have experienced a dramatic disruption of their daily routines as schools and universities have closed, consequently significantly reducing opportunities for social relationships, which are crucial in this specific phase of development. Research shows that more than a third of young adults reported high levels of loneliness, and nearly half of 18–24-year-olds felt lonely during the lockdown. Considering these data and the existing correlation between isolation, reported feelings of loneliness and mental health, a review of 63 studies has highlighted the effective consistency of this association concerning the future development of depression and post-traumatic stress disorder [7].

In addition, during the lockdown, all educational institutions had to respond rapidly to the need to change teaching in order to make it accessible online. This shift also resulted in unexpected expenses, which psychologically affected students’ well-being. Although online instruction (so-called e-learning) has proven to be a valuable alternative to face-to-face classes during the lockdown, and students have agreed with its necessity, they also displayed a negative attitude toward the new learning condition. This negative perception may have contributed to the psychological distress lamented by most young students [3]. In addition, several studies have pointed out high anxiety levels in students caused by the lack of pleasure and satisfaction in taking courses with no interaction with peers [8].

Therefore, to extend the discussion about how meaningful the interpersonal experience is for such age groups and how deleterious this moment in history has been, one must remember the significance of the peer group’s role in the formation of the adolescent’s identity, and in their general mental health and well-being. Peer support is known to be crucial in constructing more stable self-esteem, having a higher self-efficacy, and promoting and shaping the formation of coping mechanisms, such as the ability to ask for help and the maintenance of an internal locus of control. Such a mental disposition contributes to one’s feeling of control over the events [9].

Emotional regulation refers to a series of processes, dependent on an individual’s goals, that consist of the mitigation, intensification and/or maintenance of a given emotion [10,11]. These regulation processes can be automatic or controlled, conscious or unconscious; in either case, they involve dynamic changes that last over time [11]. With the development of proper emotional regulation, a person can perform more adaptive behaviors and tolerate frustrating experiences. Focusing on the pandemic period, the relevance of having adequate social skills to cope during a time of isolation is also evident, with emotional regulation being one of the best predictors of an individual’s social skills. However, children and adults strive to regulate their emotions; negative emotional states (anger, fear, frustration) must be managed as the cause of primarily subjective distress that undermines the person’s overall functioning [12].

Within the population of students, emotional regulation becomes crucial in adapting behavior to school and university goals. Emotions can indeed have an influence on the cognitive learning processes from the early motivational components to the time of information retrieval required for tests and exams. Thus, it is crucial to consider students’ cognitive and emotional patterns during times of stress to prevent future failures.

Under a situation such as the pandemic lockdown, the entire population was exposed to feelings of anxiety and fear, in general, but amongst the youth population who had pre-existing vulnerabilities as well as unfavorable social conditions, the mental health risks were even more significant, precisely due to the condition of their emotional immaturity [4].

These negative responses are more likely to occur and to be more severe in the presence of certain risk factors, among which a lack of primary resources (water, food, clothing) and inadequate information about events was found by Serafini et al. [2] to be the most unfavorable. In contrast, protective factors, including psychological resilience, active social support and preventive strategies such as effective communication, appropriate psychological listening and help services, may reduce the extent of possible emotional disturbances [2].

A recent review summarized the psychological and adaptive responses of people during the outbreak of previous epidemics and climate disasters: problem-solving skills, seeking social support and maintaining a positive appreciation, for example, toward one’s country’s government and health care system have emerged as some of the most beneficial strategies for dealing with times of crisis. In contrast, approaches characterized by distraction-seeking, denial and avoidance of the situation were negatively correlated with subsequently reported levels of stress [13].

Compared with previous social crises, as in the case of natural disasters in which all members of the community congregated both physically and socially for a common purpose, the COVID-19 time was characterized by the individual’s need for isolation; this required the individual to make an unprecedented effort to remain resilient and optimistic [14].

Several studies have analyzed resilience and coping (as outcomes of the resilience response) during the current pandemic. A study conducted on the Chinese population in the early stages of COVID-19 spread confirmed the strong correlation between coping strategies and psychological well-being, showing that individuals who implemented negative coping styles showed higher levels of distress. The same study found that, among respondents, younger individuals were not only the most psychologically affected, but also those who implemented more negative strategies [15]. Other research has addressed as many compensatory behaviors enacted by young adults: alcohol abuse as a coping strategy and altered sleep patterns. Regarding alcohol abuse, an Italian study revealed a significant increase (from 0.88% to 11.3%) in the frequency of emergency room admissions for alcohol intoxication in the first time of reopening compared to pre-COVID data. A strong indication is that these numbers refer to the 13–24 age group, and almost half of the cases included 16–18-year-olds [16]. On the other hand, observing the sleep patterns of adolescents and young adults, there is a significant delay in the time of going to bed and consequently also in waking up. The underlying motivation can be traced back to the sharp increase in time spent in front of a screen and the already many hours spent following online education. To compensate for the lack of socialization and relationship opportunities, an accomplice to the intense feeling of boredom, children, adolescents, and young adults turned to social media, video games, and TV shows. However, this led to high levels of anxiety before bedtime, which resulted in altered normative patterns [6].

A further study that investigated what may be protective factors for overcoming stressful events and experiencing less psychological pressure pointed out that greater age ranks as a better protector, along with the ability to use mindfulness. In fact, older age correlates with better mindfulness, cognitive resilience and emotional balance, allowing for more optimal resilience responses and coping strategies than young adults [17].

These latest data underscore how important it is to pay attention to the mental health of younger people, as it is clear that although they have the resilience resources to cope with problems, a deep analysis of the relation between these variables is needed. Therefore, the present study aims to assess well-being, negative psychological consequences, emotional vulnerability and resilience in a sample of university students scattered throughout the Italian country through an online platform during lockdown (T0; April 2020) and during term (T1; May–June 2020). We hypothesize that (1) as resilience levels increase, levels of ill-being (measured as stress, anxiety and depression) decrease both before and after the pandemic event; (2) as resilience levels increase, emotional regulation skills increase and, indirectly, its influence on levels of ill-being (measured as stress, anxiety and depression) through emotion regulation competencies; (3) as levels of emotional regulation increase, levels of ill-being (measured as stress, anxiety and depression) decrease both before and after the pandemic event; and (4) as levels of ill-being decrease at the beginning of the pandemic event, levels after the pandemic event also decrease.

## 2. Materials and Methods

### 2.1. Participants

A convenience sample completed an on-line survey throughout a weblink. The surveys were sent in April 2020 (T0) and in May 2021 (T1). Of the 649 subjects who responded to the survey at T0, 201 were deleted because they did not complete the entire battery and one hundred because were older than 24 years. AT T1, all subjects were contacted by email asking to complete the follow-up questionnaire. Only 97 responded and completed the survey and 33 were deleted because they did not complete the entire test battery. Finally, 339 subjects were retained at T0 and 64 at T1. At baseline, 303 subjects were female. All subjects aged 18–24 years.

### 2.2. Instruments

The resilience scale for adults (RSA) is a multidimensional questionnaire consisting of 33 items aimed at measuring 6 different dimensions of resilience [18,19]. The 6 dimensions are investigated through items characterized by a semantic differential response modality based on two opposite poles reported in a 5-step scale. An example of the questions is: “When a sudden event happens…”, followed by two opposite statements, “I always find the solution” or “I feel lost/disoriented”. The instrument allows for the measurement of the following 6 scales: (1) perception of self (RSA_Pe_se; 6 items), which refers to self-esteem, self-efficacy, being determined, etc.; (2) social competence (RSA_So_co; 6 items), which refers to extroversion/energy, having a cheerful mood, being skillful in relating and engaging in new activities, having good communication skills and being flexible in social matters, etc.; (3) structured style (RSA_St_st; 4 items), which measures the ability to sustain daily routines, plan and organize, etc.; (4) family cohesion (RSA_Fa_co; 6 items), which refers to aspects related to family coherence, family warmth, measuring the amount of conflict and family climate, cooperation to support, trust and stability relative to the family, etc.; (5) social resources (RSA_So_re; 6 items), which refers to the support of friends and relatives and their ability to get intimate or provide support, etc.; and (6) perception of the future (RSA_Pe_fu; 4 items), which measures positive outlook with respect to one’s future, confidence that life will offer other opportunities, formulation of clear and concrete goals, hope and optimism for the future, etc. Italian validation of the instrument reported a Cronbach’s alpha ranging from 0.67 to 0.90 and test–retest indices ranging from 0.69 to 0.84. Scoring for each scale represents the sum of items’ responses.

The difficulties in emotion regulation scale (DERS) [20,21] contains 36 multiple-choice items ranging from 1 (almost never) to 5 (almost always) (5). The questionnaire measures individual patterns of emotion regulation through the following 6 scales: (1) lack of acceptance (DER_la_ac; 4 items), which reflects the tendency to experience negative emotions in response to a primary negative emotion, as well as the person’s difficulties in accepting the negative emotion experienced; (2) difficulty in distracting oneself from the emotion and performing alternative behaviors (DER_di_di; 4 items), which consists of items that reflect the difficulty in completing one’s work or concentrating when experiencing negative emotions because of the arousal by which they are characterized and the consequent tendency to monopolize all of the person’s attentional resources; (3) lack of confidence in one’s emotional regulation skills (DER_la_co; 8 items), which reflects the person’s level of confidence about personal abilities to manage and modulate one’s negative emotions; (4) difficulty in controlling behaviors (DER_di_co; 6 items), which includes statements that reflect difficulty in maintaining control over one’s behaviors when experiencing negative emotions; (5) difficulty in recognizing experienced emotion (DER_di_re; 5 items), which reflects the degree to which a person recognizes the emotion he or she is experiencing; and (6) reduced emotional self-awareness (DER_re_se; 3 items), which reflects emotional awareness, i.e., the degree of attention paid to one’s emotional state. The internal consistency of the different scales of the Italian version ranges from 0.74 to 0.88. Scoring for each scale represents the mean of item responses.

The perceived stress scale (PSS; [22]) is a commonly used questionnaire aimed to measure “the degree to which individuals appraise situations in their lives as stressful” [22]. The PSS is currently translated into 25 languages (see [23]). Stress is measured through 10 items which assess the degree to which individuals believe their life has been unpredictable, uncontrollable or overloaded during the previous month using a 5-point scale ranging from never (0) to very often (4). The internal consistency of the original version has reported a Cronbach’s alpha of 0.78. Scoring represents the sum of items’ responses.

The symptom check list-90-revised (SCL-90-R) [24,25] is a 90-item self-report inventory reproducing a series of psychiatric symptomatology under 9 dimensions and 3 global indices and assessing the degree of distress experienced during the past 7 days through a 5 step-scales ranging from not at all (0) to extremely (4). Only the depression (DEP, 13 items) and anxiety (ANX, 10 items) scales were used for this study. Scoring for each scale represents the mean of item responses.

### 2.3. Data Analysis

Data were cleaned, coded and scored using Excel and Jamovi [26]. The reported confidence intervals were 95%. Normality assumptions were assessed by QQ plots and density histograms, and scatter plots were used to assess homoscedasticity and autocorrelation. Multicollinearity was assessed through variance inflation factors (VIFs). 

SEM was used to model the effect of resilience and emotion regulation difficulty on ill-being (depression, anxiety, stress) measured at baseline and at follow-up and were also loaded onto a global latent factor of “Mental Health” to control for any shared variance across variables [27].

SEM (structural equation modelling) allows for multivariate analysis, using a complex set of regression analyses to determine the relationship between measured variables and previously defined latent variables based on multiple observed variables. It is possible with SEM to analyze the interrelationship between multiple variables and to draw conclusions of causality among them [28].

The SEM was designed based on the literature, and variables were categorized into latent and observed. The SEM model was composed of four latent variables, one of which were defined as exogenous and the remaining three as endogenous variables. The exogenous latent variable was defined “res” and composed of all the six observed variables which explain the RSA. The three endogenous variables were defined as “der”, composed by all the six observed variables which explain the DERS, and “mlt_hlt_pr” and “mlt_hlt_ps”, respectively, composed by the observed ANX and DEP scale of the SCL-90-R and PSS measured at T0 (for mlt_hlt_pr) and at T1 (mlt_htl_ps).

The model fit was then evaluated, and any changes were made based on the modification indices (correlating observed variables with an estimate greater than 10) and we eliminated relationships whose parameter estimates were less than 0.50 depending on how much the fit indices improved or based on the authors’ intuitions.

The following criteria for fit indices were referred to for model performance: χ^2^, root mean square error approximation (RMSE), standardized root mean square residual (SRMR), comparative fit index (CFI), Tucker–Lewis index (TLI) and goodness of fit (GFI) [29,30].

## 3. Results

Normality tests and Mardia tests did not reveal skewness or Kurtosis tendencies (Skewness: χ^2^ = 858; df = 816; *p* = 0.150; Kurtosis: z = −0.750; *p* = 0.453); thus, the maximum likelihood method was used for the estimation of SEM parameters [31]. Table 1 reports the scores of each observed variable of the model.

As can be seen in Table 1, stress levels remain high at both T0 and T1, although decreasing significantly at T1. Anxiety and depression levels remain roughly unchanged at an average high level. In comparison with the reference values of the original Italian version [20,21], all DERS scales presented medium values except for DER_la_co and DER_di_co, which reported higher values. Moreover, in comparison with the reference values of the original Italian version [18,19], all RSA scales presented medium values. In general the sample subjects exhibit a good level of resilience and emotional regulationat T0, except for lack of confidence and difficulty in controlling behavior with regard to emotions.

**Figure 1:**
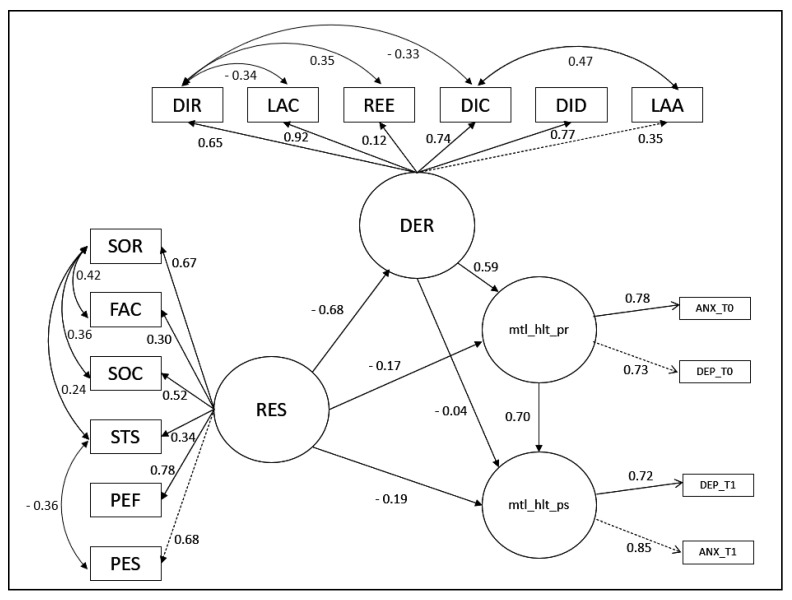
SEM model. Legend: res: latent exogenous variable measuring resilience; der: latent endogenous variable measuring difficulty in emotion regulation; mtl_hlt_pr: latent exogenous variable measuring ill-being at T0; mtl_hlt_ps: latent exogenous variable measuring ill-being at T1; ANX_T0: anxiety scale of the SCL-09-R at T0; ANX_T1: anxiety scale of the SCL-09-R at T1; DEP_T0: depression scale of the SCL-09-R at T0; DEP_T1: depression scale of the SCL-09-R at T1; LAA: lack of acceptance scale of the DERS; DID: difficulty in distracting scale of the DERS; LAC: lack of confidence scale of the DERS; DIC: difficulty in controlling behavior scale of the DERS; DIR: difficulty in recognizing emotions scale of the DERS; REE: reduced emotional self-awareness scale of the DERS; PES: perception of self scale of the RSA; PEF: perception of the future scale of the RSA; STS: structured style scale of the RSA; SOC: social competence scale of the RSA; FAC: family cohesion scale of the RSA; SOR: social resources of the RSA.

After assessing for modification indices, parameter estimation and covariance of residuals, the model showed the following fit index after: χ^2^ = 109 (df = 89, *p* = 0.070), RMSEA = 0.059 [0.00, 0.094], SRMR = 0.081, CFI = 0.95, TLI = 0.93, GFI = 0.99. Moreover, the PSS scale was removed from the latent ill-being variables both at T0 and T1 to achieve the good fit indices and fit the model. The linear relationships between variables and beta coefficients in the model are presented in Figure 1.

The model presents a strong negative relationship between resilience and emotional regulation, demonstrating how as resilient capacity increases, emotional difficulty decreases. Resilience, moreover, presents a negative, although not very strong, relationship with ill-being at time T0 and T1, showing how high levels of resilience at T0 predict low levels of ill-being at T0 and T1. Emotional regulation skills at T0 seem to have greater weight in reducing ill-being at T0, but not at T1. The interesting aspect of the model is related to the indirect relationship according to which levels of resilience influence the levels of ill-being at T1 by reducing them through an indirect relationship with the variable related to the emotional regulation of ill-being.

As can be seen from Table 2, all subscales of the RSA observed variables have a strong significant relationship with the latent resilience factor (res). Moreover, anxiety and depression scales at T0 and T1 report strong relation with the respective latent variables, and the latent variable DER shows strong relationships with its observed variables except for the subscale DER_se_re.

Reliability indices and the average of explained variance are reported in Table 3. As can be seen, all latent variables have good reliability indices. This is indicative of good internal consistency of measurement, showing that all observed variables tend to measure the reference construct coherently.

## 4. Discussion

This paper aimed to explore the impact of resilience and emotional regulation on negative psychological outcomes related to distress in university students during the COVID-19 pandemic. Our first hypothesis, which states that as the level of resilience increases at T0, ill-being decreases both before and after the pandemic event, was confirmed. The second hypothesis, which states that as resilience increases, emotion regulation skills increase as, indirectly, does its influence on levels of ill-being through emotion regulation competencies, was partially confirmed. In fact, emotion regulation has influenced the reduction of ill-being level at T0 but not at T1. The third hypothesis, stating that as levels of emotional regulation increase, levels of ill-being decrease both before and after the pandemic event, was partially confirmed. Indeed, emotion regulation competencies have only influenced ill-being at T1. The fourth hypnosis, which states that as levels of ill-being decrease at T0, levels of ill-being at T1 also decrease, was confirmed.

The results demonstrate a tendency for the students to be aware of and in control of negative emotionality, although they still experienced the negative consequences. Resilience, moreover, was a critical protective factor in reducing the negativity of the emotional experience, both at T0 and T1. This is in line with what has been reported in a recent review that synthesized people’s psychological and adaptive responses during the outbreak of previous epidemics and climate disasters. The results of this review showed that the most effective coping strategies were related to the use of problem-solving skills, the ability to seek social support and the maintenance of positive evaluation about one’s country’s institutions. Avoidance, denial and distraction-seeking strategies were correlated with high levels of stress [13].

Multiple coping strategies (e.g., behavioral activation, acceptance-based coping, mindfulness practice, loving-kindness practices) are essential to decrease stress and promote resilience and recovery. These strategies may be especially effective because they help individuals make meaning, build distress tolerance, increase social support, foster a view of our deep human interconnectedness and take goal-directed value-driven actions during the COVID-19 pandemic [14].

Emotional regulation also appears to mediate between resilience and mental ill-being, but only during the lockdown, revealing itself to be a more incisive variable in the pre-evaluation of ill-being compared to the T1 evaluation. Good emotional management has been reported to be part of the development of problem-solving skills, which are critical to having effective cognitive performance when it comes to inhibition and, for example, in substituting long-term goals for immediate incentives [32], a skill that may have proved very useful in lockdown. Therefore, emotional regulation activation seems consistent, especially in the early part of the lockdown. This evidence is coherent with an Italian study that targeted younger people [5]. This study focused on adolescents and their narratives toward the lockdown period to investigate their ability to positively or negatively re-read the new context in which they found themselves living. Initially, the results revealed that adolescents were more likely to report their experiences in a negative light. This finding is not a surprise since it was already known in the literature that negative narratives are longer and more consistent since they reflect the individual’s need to process more profoundly traumatic and chaotic experiences to make sense of them [33]. The negative aspects highlighted focused on the limitation of autonomy and the difficulty in expressing and discovering one’s new identity. Still, the youths also proved in many cases to re-read their condition positively, showing evidence of being able to use coping resources. These reinterpretations focused on the opportunity to re-discover oneself through new moments conducive to introspection, i.e., by finding oneself, and sharing the same spaces with family for significantly prolonged periods. Both aspects mentioned above were considered part of an overall process of personal growth and helped maintain the youths’ well-being [5]. 

Finally, distress seems to be more related to symptoms such as anxiety and depression rather than to a general condition of psychological stress. Indeed, to fit the model adequately, stress, as measured by the PSS, was deleted.

The ability of resilient resources to reduce distress is thus, more plausibly, related to the symptomatic experience of distress (such as anxiety and depression) rather than to the psychological component of stress. Emotional regulation plays an important mediating role in this relationship.

## 5. Conclusions

The results obtained in this study show that psychological resilience is among the protective factors that can reduce the extent of possible emotional disturbances resulting from adverse conditions such as a pandemic outbreack. This leads to the conclusion that the implementation of appropriate psychological listening and helping services and an implementation of telemedicine to enable counselling and to carry on new or already initiated therapies, especially with fragile and vulnerable people such as adolescents and young psychological adults, is essential [2]. Furthermore, smartphone apps for helping support [34,35], active social networks, dedicated blogs and forums should be implemented in order to reduce social isolation and loneliness as well as allow specific populations to successfully communicate with their loved ones [2,36], being wary of inducing the experience of feeling so-called “forced empathy” (or “to be forced to feel”) or internet addiction [37]. This aspect has been also emphasized by the use of technological devices which have led to a depersonalization of relationships, forcing the sense of closeness, at least virtually [10].

To reduce negative consequences, such as alcohol and drug abuse, specific interventions using digital and new technologies could be applied, such as those focused on reducing the craving for substances [38,39,40] or addressed to enhance resilience and protective factors [41]. Taking into consideration the population of young students, the role of emotional regulation becomes crucial in order to adapt behavior to school and college goals since emotions influence the cognitive process of learning from the component of initial motivation to the time of information retrieval for tests and exams. Therefore, it is crucial for educators and teachers to take into account students’ cognitive and emotional patterns during stressful moments to prevent future failures.

Despite the important results obtained in our study, it is important to underline some limits. First, sample numerosity is relatively limited, as is the ratio of males to females. Future studies could replicate the same results on a more balanced sample. In addition, our study has the limitation of not being re-evaluated at a second follow-up (T2). Finally, the selection of subjects may have been biased in that the convenience sample was collected from students directly attending the online classes of the authors of this study.

## Figures and Tables

**Table 1:** Mean, median and standard deviation for each of the variable’s models.

	N	Mean	Median	Standard Deviation	Minimum	Maximum
PSS	339	24.6	24.0	3.95	15.0	38.0
post_PSS	65	20.2	19.0	4.63	0.00	30.0
AXS	339	1.77	1.50	0.971	0.00	4.00
post_ANX	64	1.75	1.50	1.07	0.00	4.00
DEP	339	1.67	1.50	1.07	0.00	4.00
post_DEP	64	1.67	1.50	0.981	0.00	4.00
DER_la_ac	339	1.86	1.50	0.939	1.00	5.00
DER_di_di	339	2.97	3.00	1.05	1.00	5.00
DER_di_co	339	2.05	1.83	0.911	1.00	5.00
DER_re_se	339	2.56	2.33	1.02	1.00	5.00
DER_la_co	339	2.49	2.38	0.615	1.38	4.50
DER_di_re	339	2.39	2.20	0.839	1.00	4.60
RSA_Pe_se	339	19.9	20.0	4.21	9.00	29.0
RSA_Pe_fu	339	13.8	14.0	3.72	4.00	20.0
RSA_St_st	339	15.7	16.0	3.24	7.00	20.0
RSA_So_co	339	28.1	29.0	5.18	11.0	37.0
RSA_Fa_co	339	22.3	23.0	5.33	8.00	30.0
RSA_So_re	339	30.8	32.0	3.91	15.0	35.0

Legend: PSS: perceived stress scale at T0; post_PSS: perceived stress scale at T1; ANX: anxiety scale of the SCL-09-R at T0; post_ANX: anxiety scale of the SCL-09-R at T1; DEP: depression scale of the SCL-09-R at T0; post_DEP: depression scale of the SCL-09-R at T1; DER_la_ac: lack of acceptance scale of the DERS; DER_ di_di: difficulty in distracting scale of the DERS; DER_la_co: lack of confidence scale of the DERS; DER_di_co: difficulty in controlling behavior scale of the DERS; DER_di_re: difficulty in recognizing emotions scale of the DERS; DER_re_se: reduced emotional self-awareness scale of the DERS; RSA_Pe_se: perception of self-scale of the RSA; RSA_Pe_fu: perception of the future scale of the RSA; RSA_St_st: structured style scale of the RSA; RSA_So_co: social competence scale of the RSA; RSA_Fa_co: family cohesion scale of the RSA; RSA_So_re: social resources of the RSA.

**Table 2:** Measurements model.

				95% Confidence Intervals				
Latent	Observed	Estimate	SE	Lower	Upper	β	z	*p*
res	RSA_Pe_se	1.000	0.000	10.000	1.000	0.681		
	RSA_Pe_fu	1.017	0.210	0.6062	1.428	0.779	4.853	<0.001
	RSA_St_st	0.363	0.175	0.0188	0.707	0.336	2.067	0.039
	RSA_So_co	1.102	0.310	0.4938	1.710	0.521	3.552	<0.001
	RSA_Fa_co	0.573	0.268	0.0485	1.097	0.302	2.141	0.032
	RSA_So_re	1.062	0.240	0.5909	1.532	0.670	4.421	<0.001
mtl_hlt_pre	ANX	1.000	0.000	10.000	1.000	0.775		
	DEP	0.996	0.193	0.6169	1.375	0.731	5.149	<0.001
mtl_hlt_pos	post_DEP	1.000	0.000	10.000	1.000	0.722		
	post_ANX	1.291	0.254	0.7935	1.788	0.855	5.086	<0.001
DER	DER_la_ac	1.000	0.000	10.000	1.000	0.347		
	DER_di_di	2.285	0.786	0.7433	3.826	0.766	2.905	0.004
	DER_di_co	1.991	0.633	0.7510	3.231	0.745	3.147	0.002
	DER_re_se	0.367	0.419	−0.4549	1.188	0.120	0.875	0.382
	DER_la_co	1.652	0.596	0.4828	2.821	0.923	2.770	0.006
	DER_di_re	1.718	0.688	0.3696	3.067	0.649	2.497	0.013

Legend: res: latent exogenous variable measuring resilience; der: latent endogenous variable measuring difficulty in emotion regulation; mtl_hlt_pre: latent exogenous variable measuring ill-being at T0; mtl_hlt_pos: latent exogenous variable measuring ill-being at T1; ANX: anxiety scale of the SCL-09-R at T0; post_ANX: anxiety scale of the SCL-09-R at T1; DEP: depression scale of the SCL-09-R at T0; post_DEP: depression scale of the SCL-09-R at T1; DER_la_ac: lack of acceptance scale of the DERS; DER_ di_di: difficulty in distracting scale of the DERS; DER_la_co: lack of confidence scale of the DERS; DER_di_co: difficulty in controlling behavior scale of the DERS; DER_di_re: difficulty in recognizing emotions scale of the DERS; DER_re_se: reduced emotional self-awareness scale of the DERS; RSA_Pe_se: perception of self-scale of the RSA; RSA_Pe_fu: perception of the future scale of the RSA; RSA_St_st: structured style scale of the RSA; RSA_So_co: social competence scale of the RSA; RSA_Fa_co: family cohesion scale of the RSA; RSA_So_re: social resources of the RSA.

**Table 3:** Reliability indices of the SEM latent variables.

Variable	α	ω₁	ω₂	ω₃	AVE
res	0.750	0.653	0.653	0.624	0.310
mtl_hlt_pre	0.723	0.723	0.723	0.723	0.566
mtl_hlt_pos	0.761	0.774	0.774	0.774	0.635
DER	0.736	0.694	0.694	0.710	0.364

## Data Availability

No data available.
